# Supplementary material for: Parabolic avalanche scaling in the synchronization of cortical cell assemblies
Source: Nat Commun. 2023 May 3;14:2555. doi: 10.1038/s41467-023-37976-x (PMC10156782; doi:10.1038/s41467-023-37976-x)
Supplement: Supplementary file 3 — Reporting Summary [file 41467_2023_37976_MOESM3_ESM.pdf]

nature portfolio

Corresponding author(s): Dietmar Plenz
Last updated by author(s): 03/09/2023

Reporting Summary

Nature Portfolio wishes to improve the reproducibility of the work that we publish. This form provides structure for consistency and transparency in reporting. For further information on Nature Portfolio policies, see our Editorial Policies and the Editorial Policy Checklist.

Statistics

For all statistical analyses, confirm that the following items are present in the figure legend, table legend, main text, or Methods section.

n/a	Confirmed	
☐	☒	The exact sample size (n) for each experimental group/condition, given as a discrete number and unit of measurement
☐	☒	A statement on whether measurements were taken from distinct samples or whether the same sample was measured repeatedly
☐	☒	The statistical test(s) used AND whether they are one- or two-sided<br>*Only common tests should be described solely by name; describe more complex techniques in the Methods section.*
☒	☐	A description of all covariates tested
☐	☒	A description of any assumptions or corrections, such as tests of normality and adjustment for multiple comparisons
☐	☒	A full description of the statistical parameters including central tendency (e.g. means) or other basic estimates (e.g. regression coefficient) AND variation (e.g. standard deviation) or associated estimates of uncertainty (e.g. confidence intervals)
☐	☒	For null hypothesis testing, the test statistic (e.g. F, t, r) with confidence intervals, effect sizes, degrees of freedom and P value noted<br>*Give P values as exact values whenever suitable.*
☒	☐	For Bayesian analysis, information on the choice of priors and Markov chain Monte Carlo settings
☒	☐	For hierarchical and complex designs, identification of the appropriate level for tests and full reporting of outcomes
☐	☒	Estimates of effect sizes (e.g. Cohen's d, Pearson's r), indicating how they were calculated

Our web collection on statistics for biologists contains articles on many of the points above.

Software and code

Policy information about availability of computer code

Data collection	ThorImageLS and Thorsync v4.0 (Thorlabs Inc.), ScanImage Premium 2021.0.0 (Vidrio Technologies)
Data analysis	Matlab from 2019a (v9.6) to 2021a (v9.10) (Mathworks), Suite2P https://github.com/MouseLand/suite2p (motion correction, ROI identification). MLspike https://github.com/MLspike (deconvolution of 2PI GECI signals). The Deep-Interpolation github repo: https://github.com/AllenInstitute/deepinterpolation.We used version 0.1.3 and haven't updated since. The newest version is 0.1.4 and contains minor improvements that aren't necessary for our purposes. We ran the package using Python version 3.7.<br>Additional data analysis was conducted in Python 3.7 using Numpy 1.22.2 and Matplotlib 3.5.1.<br>Computer code is available at GitHub https://github.com/PlenzLab/ParabolicAvalanches/.

For manuscripts utilizing custom algorithms or software that are central to the research but not yet described in published literature, software must be made available to editors and reviewers. We strongly encourage code deposition in a community repository (e.g. GitHub). See the Nature Portfolio guidelines for submitting code & software for further information.

Data

Policy information about availability of data

All manuscripts must include a data availability statement. This statement should provide the following information, where applicable:

- Accession codes, unique identifiers, or web links for publicly available datasets
- A description of any restrictions on data availability
- For clinical datasets or third party data, please ensure that the statement adheres to our policy

The preprocessed imaging data used in this study are available in the general repository Zenodo using the following access DOI: https://doi.org/10.5281/zenodo.7703224 (Ref. (Capek, 2023). The source data for all figures and supplemental figures in this study are provided in the Supplementary Information/Source

Data file 'ParabolicAvalanches.zip'.

Field-specific reporting

Please select the one below that is the best fit for your research. If you are not sure, read the appropriate sections before making your selection.

[x] Life sciences [ ] Behavioural & social sciences [ ] Ecological, evolutionary & environmental sciences

For a reference copy of the document with all sections, see nature.com/documents/nr-reporting-summary-flat.pdf

Life sciences study design

All studies must disclose on these points even when the disclosure is negative.

Sample size	For each mouse, continuous imaging periods were kept to <30 min to reduce overt stress to the animal. Multiple imaging periods from each animal were analyzed separately and significant events were combined to obtain distributions with >300 population events. Previous estimates suggested this minimal number of events is required to obtain significant differences between randomized and actual distributions. Between 3 - 7 mice were analyzed per region and data set.
Data exclusions	Experiments were excluded when the available number of neurons was <100.
Replication	We replicated our results for each animal, different brain regions (AAC, mPFC, V1) and from two different laboratories (our own lab) and a publicly available Allen Institute data set.
Randomization	We used several different shuffle procedures to demonstrate significance of our results. We typically presented mean and standard deviation in our plots in addition to significance values at given significance level.
Blinding	Blinding was not relevant in this study as the effect was shown by shuffling/controlled randomization for each animal and recording condition.

Reporting for specific materials, systems and methods

We require information from authors about some types of materials, experimental systems and methods used in many studies. Here, indicate whether each material, system or method listed is relevant to your study. If you are not sure if a list item applies to your research, read the appropriate section before selecting a response.

Materials & experimental systems

n/a	Involved in the study	
[x]	[ ]	Antibodies
[x]	[ ]	Eukaryotic cell lines
[x]	[ ]	Palaeontology and archaeology
[ ]	[x]	Animals and other organisms
[x]	[ ]	Human research participants
[x]	[ ]	Clinical data
[x]	[ ]	Dual use research of concern

Methods

n/a	Involved in the study	
[x]	[ ]	ChIP-seq
[x]	[ ]	Flow cytometry
[x]	[ ]	MRI-based neuroimaging

Animals and other organisms

Policy information about studies involving animals; ARRIVE guidelines recommended for reporting animal research

Laboratory animals	All animals were obtained from NIH approved vendors or from our NIH inhouse animal breeding facility. Animals underwent surgery at ages > 6 weeks and imaged at ages between 7 - 10 weeks. Animals were taken from the in-house facility to the laboratory several hours before recordings and returned to the facility on the same day.<br><br>Mice were held in the facility using standard equipment from Allentown. All cages are filled with bedding, accessible water, and food. The mice are checked twice daily during the week and once daily on the weekends. Mice were kept on a reverse light cycle using a red bulb from 9am-9pm and standard white light from 9pm-9am. The temperatures and humidity are monitored and recorded daily. The humidity is kept within a range of 35%-65% and the temperature ranges from 72-74 degrees fahrenheit.
Wild animals	No wild animals were used in the study.
Field-collected samples	No field collected samples were used in the study.
Ethics oversight	All animal procedures were approved by the NIMH Animal and Use committee based on the animal study protocol LSN-09.

Note that full information on the approval of the study protocol must also be provided in the manuscript.
